# UAPF: A UWB Aided Particle Filter Localization For Scenarios with Few Features

**DOI:** 10.3390/s20236814

**Published:** 2020-11-28

**Authors:** Yang Wang, Weimin Zhang, Fangxing Li, Yongliang Shi, Fuyu Nie, Qiang Huang

**Affiliations:** 1 School of Mechatronical Engineering, Beijing Institute of Technology, Beijing 100081, China; 3120190166@bit.edu.cn (Y.W.); wonk2000@bit.edu.cn (F.L.); ylshi@bit.edu.cn (Y.S.); 3120190164@bit.edu.cn (F.N.); qhuang@bit.edu.cn (Q.H.); 2 Key Laboratory of Biomimetic Robots and Systems, Ministry of Education, Beijing Institute of Technology, Beijing 100081, China; 3 Beijing Advanced Innovation Center for Intelligent Robots and Systems, Beijing 100081, China

**Keywords:** particle filters, sensor fusion, ultra wideband technology, robot localization, robot kidnap recovery

## Abstract

Lidar-based localization doesn’t have high accuracy in open scenarios with few features, and behaves poorly in robot kidnap recovery. To address this problem, an improved Particle Filter localization is proposed who could achieve robust robot kidnap detection and pose error compensation. UAPF adaptively updates the covariance by Jacobian from Ultra-wide Band information instead of predetermined parameters, and determines whether robot kidnap occurs by a novel criterion called KNP (Kidnap Probability). Besides, pose fusion of ranging-based localization and PF-based localization is conducted to decrease the uncertainty. To achieve more accurate ranging-based localization, linear regression of ranging data adopts values of maximum probability rather than average distances. Experiments show UAPF can achieve robot kidnap recovery in less than 2 s and position error is less than 0.1 m in a hall of 40 by 15 m, when the currently prevalent lidar-based localization costs more than 90 s and converges to wrong position.

## 1. Introduction

Localization technology is subdivided into outdoor and indoor localization according to application scenarios. Global Positioning System (GPS)-based outdoor positioning services has almost matured and is widely used. However, GPS cannot achieve indoor positioning accurately due to severe occlusion. Moreover, indoor localization will bring inevitable errors to the results due to complex environmental structure, uncertain conditions, and numerous obstacles [1].

In order to cope with the state estimation of robots, localization based on probabilistic algorithms is the only effective solution currently known [2]. As the core idea of probabilistic localization, Bayesian filtering algorithm occupies an important role. In the early days, the best technology for implementing Bayesian filtering was Kalman Filter (KF), which could achieve efficient state estimation for linear Gaussian systems, but difficult to depict non-linear systems. Therefore, extended Kalman Filter (EKF) and unscented Kalman Filter (UKF) were proposed to solve the state estimation in nonlinear systems. In general, EKF and UKF perform well except systems highly non-Gaussian distributions. On this basis, PF is applied as a non-parametric filter [2], whose typical implementation is AMCL [3], which performs well in localization efficiency, stability, and accuracy, but poorly in global localization in scenarios with few features.

Indoor localization technology can be subdivided into single sensor localization and multi-sensor localization whose sensors include ultrasonic [4], infrared [5,6], vision [7], lidar, radio frequency identification (RFID) [8,9], Bluetooth [10], Wi-Fi [11] and so on. In addition, UWB [12,13] has also become a research hot-spot in recent years. Due to the limitations of a single sensor, multi-sensor combined localization is generally used in actual applications.

The odometry is a very widely used sensor in wheeled robot localization [11,14]. It has the characteristics of easy data processing, controllable accuracy, and high universality. However, because of accumulated errors, localization accuracy exists during long-term operation will gradually decrease. Thanks to the high ranging accuracy, little influence of light, and easy installation, lidar is popular in various autonomous robots [15,16,17]. However, the effective measure distance is limited, and the matching-based method has the disadvantages of high cost and low efficiency in achieving global localization. UWB related technology has made remarkable progress since it was approved for civilian application, which has the advantages of wide-ranging and no accumulated error, but with a certain drift in the localization process. At present, the accuracy of Sapphire system of Multi-spectral Solutions is under 10 cm. Salman et al. implemented UWB localization on a mobile robot, CoLORbot, for localization in indoor unknown scenarios [13].

Therefore, there are certain disadvantages when a single sensor acquires information making it difficult to achieve accurate localization, due to which different kinds of sensors are usually combined for localization [18]. White introduced the general model of data fusion in 1998 [19], and Hall et al. introduced the algorithms and strategies of data fusion in detail [20]. At present, the methods employed to multi-sensor fusion localization generally include Bayesian based methods [3,11] and neural network methods [21,22]. There are numerous data fusion methods based on the multi-Bayesian estimation. The Kalman Filter (KF), a kind of Gaussian filter, is a recursive filter for linear systems. For non-linear systems, there are two types, Extended Kalman Filter (EKF) and Unscented Kalman Filter (UKF). In general, KF can complete data fusion well, but when it’s hard to find out system models, there are cases of low real-time performance and reliability. Numerous non-parametric filters are based on the Monte Carlo Localization proposed by Fox et al, which is a non-parametric filter method based on Bayesian estimation [23]. Valerio Magnago et al. combined odometry and UWB information with UKF [14]. Peng Gang et al. added an additional Gaussian-Newton-based scan-match step on the basic of AMCL, improving the localization accuracy in complex and unstructured environments [24]. In  [25], sensors node’s movement dynamics and the measurements of it’s velocity and the received signal strength (RSS) of the radio signal from above-ground relay nodes are used to achieve localization, using corresponding algorithms based on KF for different scenarios. The idea that one supervisor work as planer and the other supervisor improves the result supports the idea of this article [26]. With the development of machine learning, neural networks have attracted more attention as a new data fusion method. J.Wang et al. used BP neural network to estimate the GPS sampling time and performed subsequent data fusion [27].

In this paper, we focus on achieving robust robot kidnap detection and recovery based on PF localization, where accurate global proposal distribution, provided by ranging-based localization in UAPF, is necessary. In this case, adaptive estimation of the probability of robot kidnap is feasible with the criterion KNP, proposed to measure the probability of robot kidnap. To solve the problem of false identification of robot kidnap, robot kidnap recovery is triggered only if the uncertainty of the particle swarm is high enough, due to which the reliability of robot kidnap detection increases. Besides, for more accurate ranging-based localization, the double-sided two-way (DSTW) is used in ranging-based localization, in which Jacobin matrix is used to get the position error [2]. UAPF could make up for the deficiencies of global localization and robot kidnap recovery of PF and achieve accurate localization in open scenarios with few features. The contributions of this work are as follows:

1. An improved PF-based localization algorithm is proposed, which could achieve robust kidnap detection and pose error compensation.
2. A novel criterion named KNP is proposed to indicate the probability of robot kidnap, based on the inconsistency of two pose distribution.
3. An adaptive covariance matrix ameliorates the reliability of UAPF, which is provided by the improved proposal distribution with UWB information.

The rest of this paper is outlined as follows. In Section 2, we introduce the theoretical basis and the detailed system overview. In Section 3, pre-experiments are conducted to decrease ranging error and improve localization accuracy of UWB, after which experiments are presented to illustrate improvements of this method proposed in this paper. Finally, we highlight some conclusions.

## 2. Materials and Methods

**Figure 1:**
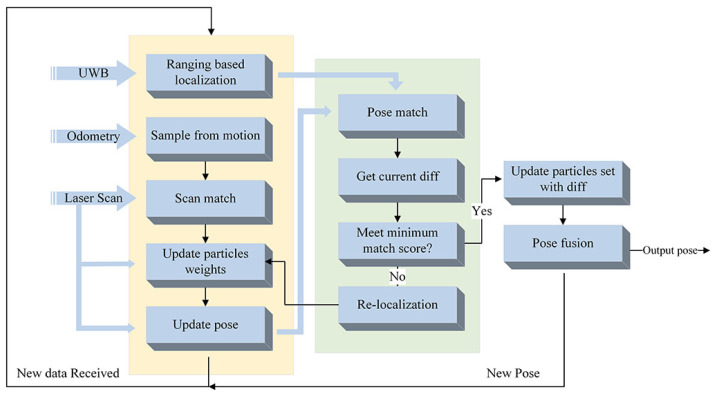
Framewrok of UAPF proposed in this paper.

UAPF is an improved PF-based localization method with adaptive robot kidnap detection and efficient kidnap recovery. This method mainly consists of PF-based localization [23], ranging-based localization and adaptive robot kidnap detection. In PF-based localization, 2D laser-scan is utilized to weight particles sampled around odometry pose. Adaptive robot kidnap detection focus on measuring how similar two poses are, and output an error transform matrix and KNP, which is the criterion to judge whether robot kidnap occurs. Besides, a supplementary adaptive update for particles uncertainty is conducted in this part, decreasing the error caused by fixed lidar measurement. The framework of UAPF is shown in Figure 1 and Algorithm 1.
**Algorithm 1:** UAPF($x_{t-1}$,$r_{i,t}$,$u_{t}$,$z_{t}$).1:$x_{r,t}=Ranging\_Based\_Localization\left(r_{i,t}\right)$2:$x_{p,t}=PF\_based\_Localization\left(x_{t-1},\;u_{t},\;z_{t},\;\Sigma_{p}\right)$3:Get KNP according to distributions of $x_{r,t}$ and $x_{p,t}$4:**if**KNP>threshold**then**5:    $x_{t}=Pose\_Fusion\left(x_{p,t},\;x_{r,t},\;\Sigma_{r}\right)$6:**else**7:    **if** $\Sigma_{p}\gg \Sigma_{r}$ **then**8:        Re-localization:    $x_{t}∼N\left(\mu_{r},\;\Sigma_{p}+\Sigma_{r}\right)$9:    **else**10:        $x_{t}=Pose\_Fusion\left(x_{p,t},\;x_{r,t},\;\Sigma_{r}\right)$11:    **end if**12:**end if**13:Update $\Sigma_{p}$ according to KNP and pose difference14:**return**$x_{t}$

Where $x_{t-1}$ is the fused pose in time t−1, $r_{i,t}$ is the distance between the robot and the $Anchor_{i}$, $u_{t}$ is the movement of the robot, and $z_{t}$ is the observing information from ranging-based localization. $x_{p,t}$ and $x_{r,t}$ are from PF-based localization and ranging-based localization separately. Similarly, $\Sigma_{p}$ and $\Sigma_{r}$ are the covariance describing the degree of pose dispersion. The threshold is a constant to determine whether trigger the re-localization process. According to our empirical data, 0.67 is a good choice to achieve good results. $N(\mu_{r},\;\Sigma_{p}+\Sigma_{r})$ is the two-dimensional Gaussian distribution with mean $\mu_{r}$ and covariance $\Sigma_{p}+\Sigma_{r})$. $x_{t}$ is the fused pose used to describe the accuracy of UAPF.

### 2.1. PF-Based Localization

Original PF-based localization is improved in this paper, whose main idea is to choose particles with high weight sampled around the pose calculated according to odometry information, as shown in Algorithm 2, which is obtained by substituting last fused pose, the increment of robot movement, environment observations and corrected particles covariance into PF.

At every moment, we get current pose with differential odometry model (line 2), after which particles are sampled following $N(\mu_{p},\Sigma_{p})$ (line 5). In this step, the use of $\Sigma_{p}$, corrected in (21) gives a more reasonable proposal distribution, which could adaptively adjust the size of particle swarms according to last pose error, and improve the rationality and reliability of PF-based localization. For every particle, the measurement model is applied in line 6 to weigh the importance according to the matching degree to the current local environment. Candidate particle swarm ${\bar{X}}_{t}$ covers particles’ poses and corresponding weights. In the update stage, re-sample is conducted, where particles with high weights are much more possible to be sampled than ones with low weights. Finally, $x_{p,t}$ is got and input into pose fusion method (line 10 in Algorithm 1).
**Algorithm 2** Improved PF-based Localization ($x_{t-1}$,$u_{t}$,$z_{t}$,$\Sigma_{p}$).1:$X_{t}$, ${\bar{X}}_{t}=\phi$2:$x_{odom}$ = $Motion\_model(u_{t},x_{t-1})$3:Set *m* as the number of particles should be sampled4:**for** i = 0 to m **do**5:    $x_{t}^{i}$ = $Sample\_model(x_{odom},\Sigma_{p})$6:    $w_{t}^{i}$ = $Scanmach\_model(z_{t},x_{t}^{i},map)$7:    $\bar{X_{t}}$ += $<x_{t}^{i},w_{t}^{i}>$8:**end for**9:**for** i = 0 to m **do**10:    Draw $x_{t}^{i}$ from $\bar{\chi_{t}}$ with probability $\propto w_{t}^{i}$11:    $X_{t}$ += $x_{t}^{i}$12:**end for**13:$x_{p,t}$ = $Mean\left(X_{t}\right)$14:**return**$x_{p,t}$

Where $X_{t}$ is the set to store the pose and covariance of particles of whom ${\bar{X}}_{t}$ is the candidate particle swarm. The two are set to the empty set, ϕ. Pose of every particles, $x_{t}^{i}$, is sampled around $x_{odom}$, calculated according to robot movement and last robot pose, and $w_{t}^{i}$ is the corresponding weight.

### 2.2. Ranging-Based Localization

**Figure 2:**
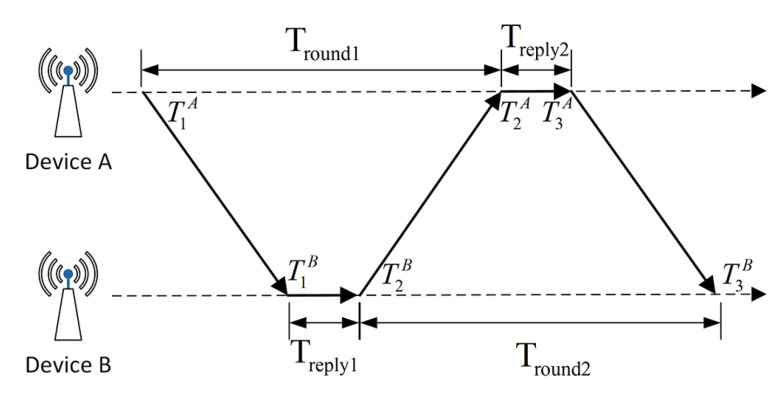
The data packet is sent 3 times in DSTW.

DSTW ranging method is used in the ranging-based localization method. The schematic diagram is shown in Figure 2. Two axes respectively indicate the time axis of device A and device B.

The predicted value of flight time ${\hat{T}}_{prop}$ can be expressed as

(1) $${\hat{T}}_{\mathrm{prop}}=\frac{T_{\mathrm{round}1}\times T_{\mathrm{round}2}-T_{\mathrm{reply}1}\times T_{\mathrm{reply}2}}{T_{\mathrm{round}1}+T_{\mathrm{round}2}+T_{\mathrm{reply}1}+T_{\mathrm{reply}2}}$$

and

(2) $$\begin{array}{cc} \; & T_{\mathrm{round}1}=T_{2}^{A}-T_{1}^{A} \end{array}$$

(3) $$\begin{array}{cc} \; & T_{\mathrm{round}2}=T_{3}^{B}-T_{2}^{B} \end{array}$$

(4) $$\begin{array}{cc} \; & T_{\mathrm{reply}1}\;=T_{2}^{B}-T_{1}^{B} \end{array}$$

(5) $$\begin{array}{cc} \; & T_{\mathrm{reply}2}\;=T_{3}^{A}-T_{2}^{A} \end{array}$$

where $T_{\mathrm{round}}$ refers to the time from sending a packet to receiving it, and $T_{\mathrm{reply}}$ refers to the time of data processing for a single device. In this way, the error of flight time can be expressed as

(6) $$T_{\mathrm{error}}={\hat{T}}_{\mathrm{prop}}\times \left(1-\frac{e_{A}+e_{B}}{2}\right)$$

where $e_{A}$ and $e_{B}$ refer to the ratio of actual frequency to the rated value of devices A and B. DSTW can solve the problem of time synchronization in some degree, improving the ranging accuracy, which is returned in millimeters.

**Figure 3:**
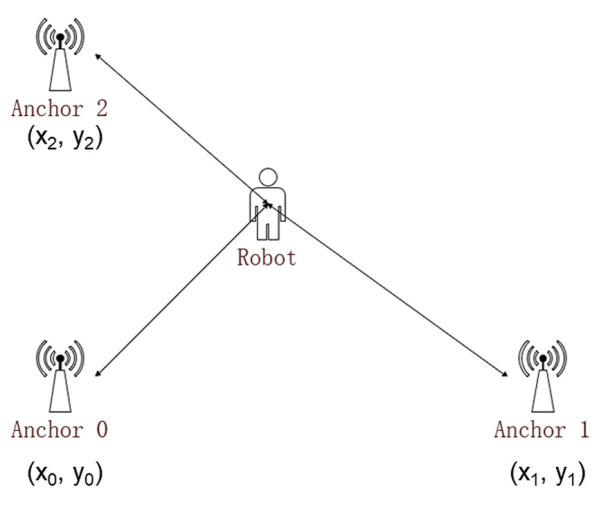
The model to find robot position. Given the positions of anchors and the distances between the some anchor to the robot, the position of robot could be calculated.

Having got ranges between some anchor and the tag, triangulation is used to calculate the robot position, $\left(x,\;y,\;z\right)$, shown in Figure 3 and Equation (7).

(7) $$\left\{\begin{array}{c} {\left(x_{0}-x\right)}^{2}+{\left(y_{0}-y\right)}^{2}+{\left(z_{0}-z\right)}^{2}=\rho_{0}^{2}\\ {\left(x_{1}-x\right)}^{2}+{\left(y_{1}-y\right)}^{2}+{\left(z_{1}-z\right)}^{2}=\rho_{1}^{2}\\ {\left(x_{2}-x\right)}^{2}+{\left(y_{2}-y\right)}^{2}+{\left(z_{2}-z\right)}^{2}=\rho_{2}^{2} \end{array}\right.$$

To cut down the cost of computation, $Anchor_{0}$ is set as the original point, with $Anchor_{1}$ set on the x-axis and $Anchor_{2}$ set on the y-axis, and all three anchors are at the same height $z_{h}$. In Equation (8), $\rho_{i}$ expresses measured distance between the tag and $Anchor_{i}$, and ($x_{i}$, $y_{i}$, $z_{i}$) refer to the position of $Anchor_{i}$.

(8) $$\left\{\begin{array}{c} x=\frac{\rho_{0}^{2}-\rho_{1}^{2}+x_{1}^{2}}{2x_{1}}\\ y=\frac{\rho_{0}^{2}-\rho_{2}^{2}-x^{2}+{\left(x_{2}-x\right)}^{2}+y_{2}^{2}}{2y_{2}} \end{array}\right.$$

Equation (8) finally shows the position of the robot in UWB coordinate system. However, the positioning accuracy cannot be estimated directly, so the detection-correction link is added, for which the error of position $\left(dx,\;dy,\;dz\right)$ is converted from the distance error using Jacobian.

As a real-time ranging-based positioning technology, positioning with UWB has no cumulative error, but the covariance varies greatly among different hardware. Therefore, the distance errors between the robot and anchors are used to derive the coordinate error to achieve accurate position estimation.

The distance between the robot and a certain anchor ${\hat{\rho }}_{i}$ can be expressed as

(9) $${\hat{\rho }}_{i}=\sqrt{{\left(x_{i}-x\right)}^{2}+{\left(y_{i}-y\right)}^{2}+{\left(z_{i}-z\right)}^{2}}$$

Assuming that the robot coordinates $\left(x,\;y,\;z\right)$ are known, the ranging error of UWB is easily obtained as

(10) $$\Delta \rho_{i}=\rho_{i}-\sqrt{{\left(x_{i}-x\right)}^{2}+{\left(y_{i}-y\right)}^{2}+{\left(z_{i}-z\right)}^{2}}$$

To obtain the coordinate error ${\left(\begin{array}{ccc} dx & dy & dz \end{array}\right)}^{T}$, Equation (10) is derived.

(11) $$d\rho_{i}=\frac{\left(x_{i}-x\right)dx+\left(y_{i}-y\right)dy+\left(z_{i}-z\right)dz}{\sqrt{{\left(x_{i}-x\right)}^{2}+{\left(y_{i}-y\right)}^{2}+{\left(z_{i}-z\right)}^{2}}}$$

By introduceing Equation (9) into Equation (11). We obtain Equation (12).

(12) $$d\rho_{i}=\frac{\left(x_{i}-x\right)dx+\left(y_{i}-y\right)dy+\left(z_{i}-z\right)dz}{\rho_{i}}$$

Therefore, by converting Equation (12) into a matrix representation, a differential matrix from the coordinate error to the distance error can be obtained. Equation (14) are used to get $\Sigma_{r}$, which is used in Algorithms 1 and 3.

(13) $$\left[\begin{array}{c} d\rho_{0}\\ d\rho_{1}\\ d\rho_{2} \end{array}\right]=\left[\begin{array}{ccc} \frac{x_{0}-x}{\rho_{0}} & \frac{y_{0}-y}{\rho_{0}} & \frac{z_{0}-z}{\rho_{0}}\\ \frac{x_{1}-x}{\rho_{1}} & \frac{y_{1}-y}{\rho_{1}} & \frac{z_{1}-z}{\rho_{1}}\\ \frac{x_{2}-x}{\rho_{2}} & \frac{y_{2}-y}{\rho_{2}} & \frac{z_{2}-z}{\rho_{2}} \end{array}\right]\left[\begin{array}{c} dx\\ dy\\ dz \end{array}\right]=T_{tran}\;\left[\begin{array}{c} dx\\ dy\\ dz \end{array}\right]$$

(14) $$\left[\begin{array}{c} dx\\ dy\\ dz \end{array}\right]=T_{tran}^{-1}\left[\begin{array}{c} d\rho_{0}\\ d\rho_{1}\\ d\rho_{2} \end{array}\right]$$

### 2.3. Robot Kidnap Detection and Recovery

To address the problem of global localization and robot kidnap detection in traditional PF-based localization methods [23], we propose a novel criterion, KNP, which is measured according to the distribution of particles, $X_{t}$, and the pose of ranging-based localization, $x_{t}$, where *t* represents values at the time *t*.

In update phase (the green rectangle in Figure 1), a match-based measurement method is conducted. We assume both PF-based poses, $x_{p}$, and ranging-based poses, $x_{r}$, follow 2D Gaussian distribution.

(15) $$\begin{array}{cc} \; & x_{p}∼N\left(\mu_{p},\Sigma_{p}\right) \end{array}$$

(16) $$\begin{array}{cc} \; & x_{r}∼N\left(\mu_{r},\Sigma_{r}\right) \end{array}$$

where $\mu_{p}$ means the center pose of PF-based localization and $\mu_{r}$ means the center pose of ranging-based localization. And the variance $\Sigma_{p}$ and $\Sigma_{r}$ presents how large sizes of particle swarms are. Moreover, $\Sigma_{r}$ is assumed to be only related to the distance between anchors and the robot because the ranging results are corrected in the range of 3 m to 20 m, and the system is used in unobstructed scenarios for  UWB, without Non-Line-of-Sight, NLOS.

(17) $$\Sigma_{r}^{{}^{\prime }}=\frac{\Sigma_{r}}{\alpha^{n}}$$

where α refers to attenuation coefficients, and *n* is the number that ranging distance over 20 m.

Moreover, to measure the possibility of robot kidnapping, a novel criterion called KNP is introduced into UAPF, expressing the difference between two kinds of localization methods. In Equation (18), expectations and covariance matrices are substituted to get the Wasserstein distance between two localization methods.

(18) $$\begin{array}{c} S=W^{2}\left(x_{p},x_{r}\right)={∥\mu_{p}-\mu_{r}∥}^{2}+tr\left[\Sigma_{p}+\Sigma_{r}^{{}^{\prime }}-2{\left(\Sigma_{p}^{1/2}\Sigma_{r}^{{}^{\prime }}\Sigma_{p}^{1/2}\right)}^{1/2}\right] \end{array}$$

Then, in Equation (19), KNP is to measure the possibility of robot kidnapping, as shown in line 5 of Algorithm 1 and Equation (21). Generally, the smaller KNP is, the more possible robot kidnap occurs, and it could maintain a relative score of 0.8 with normal operating conditions.

(19) $$KNP=\lambda (p_{1}S^{4}+p_{2}S^{3}+p_{3}S^{2}+p_{4}S^{1}+p_{5})$$

### 2.4. Particles Update for Pose Tracking

In traditional PF-based localization [3], pose error is measured by the variance of particles swarm, but sensor noise of odometry and lidar is regarded as fixed parameters, which ruins the accuracy of localization to some degree.

In general, when the robot moves from $x_{t-1}$ to $x_{t}$, with the odometry movement *u* and the map *m*, we can obtain the probability distribution of the robot pose. The combined localization of lidar and odometry is robust in most cases. However, lidar can only reduce the accumulated error of the odometry, rather than eliminates it. Therefore, UWB is introduced to eliminate the accumulative error of the system. Therefore, the probability distribution of $x_{t}$ can be expressed as (20).

(20) $$\begin{array}{c} p\left(x_{t}|x_{t-1},u,m,z\right)=\eta p\left(z|x_{t},m\right)p\left(x_{t}|x_{t-1},u\right)=\eta \prod_{j}p\left(z_{j}|x_{t},m\right)p\left(x_{t}|x_{t-1},u\right)\\ =\eta \left(z_{uwb}|x_{t}\right)p\left(z_{lidar}|x_{t},m\right)p\left(x_{t}|x_{t-1},u\right) \end{array}$$

where η is the normalization constant, $p(x_{t}|x_{t-1},u)$ expresses the odometry pose calculated by robot motion, $p\left(z_{lidar}|x_{t},m\right)$ expresses lidar likelihood domain model and $p(z_{uwb}|x_{t})$ is measured by ranging-based localization sub-process.

KNP could adaptively measure localization accuracy to some degree, but not enough for real-time pose tracking. Therefore, the euclidean distance between the results of two localization methods is taken into account to update $\Sigma_{p}$.

(21) $$\begin{array}{c} \Sigma_{p}\leftarrow \Sigma_{p}+\left[\begin{array}{ccc} \frac{\Delta x^{2}}{KNP} & & \\ & \frac{\Delta y^{2}}{KNP} & \\ & & 0_{\left(4,4\right)} \end{array}\right] \end{array}$$

where $\Sigma_{p}$ represents the covariance of PF-based localization. $\Delta x^{2}$ and $\Delta y^{2}$ are pose differences between the two localization methods. In (21), only position error is to update because of the low reliability of the yaw in ranging-based localization.

### 2.5. Pose Fusion

As mentioned above, the UWB position has large uncertainty, which is manifested as positioning results jumping around the real value. Therefore, the fusion of PF-based poses and UWB poses is conducted, to improve the accuracy, as shown in Algorithm 3.

Firstly, fusion starts with UWB pose as initial pose, for solving global localization. In every time *t*, PF-based localization is regarded as predictive pose, ${\hat{x}}_{t}$. In update stage, the result of ranging-based localization, $x_{r,t}$ and $\Sigma_{r}$ is used. Due to the high frequency (20Hz) of ranging-based localization, we use sliding window for the average pose value, especially for $\theta_{r}$.
**Algorithm 3** Pose Fusion ($x_{p,t}$,$x_{r,t}$,$\Sigma_{r}$).1:**if** t-1 = 0 **then**2:    Initialize ${\hat{x}}_{t}$ with $x_{r,t}$3:**else**4:    Predict: ${\hat{x}}_{t}$ = $x_{p,t}+\delta_{p}$5:    Update: $x_{t}=Fusion\_Method\;\left({\hat{x}}_{t},x_{r,t},\Sigma_{r}\right)$6:**end if**7:**return**$x_{t}$

Where $\delta_{p}$ is the noise compensation which obeys Gaussian distribution. $\theta_{r}$ is the yaw of the ranging-based localization pose.

## 3. Experiments and Results

### 3.1. Experimental Scenario and Platform

**Figure 4:**
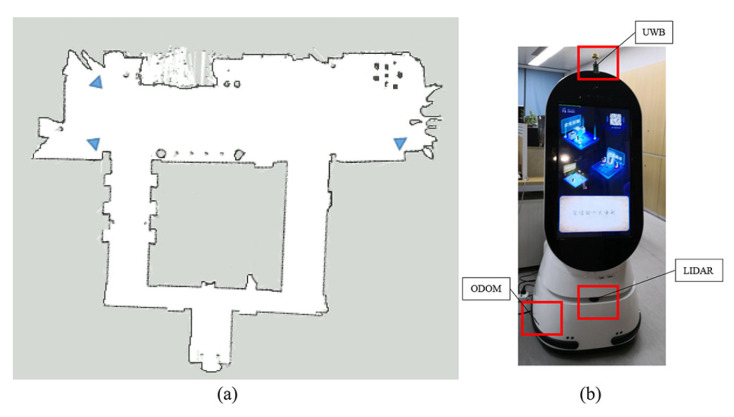
(**a**) The top half of the scenario is a hall of 40 by 15 m. The three triangles are poses of anchors. (**b**)The robot platform is equipped with odometry, lidar, and UWB.

Figure 4a shows the experimental scenario of this paper. The robot platform used in the experiment is shown in Figure 4b.

### 3.2. Ranging Experiments

Experiments on the ranging results of UWB are done, whose purpose is to decrease the ranging error caused by the hardware.

**Figure 5:**
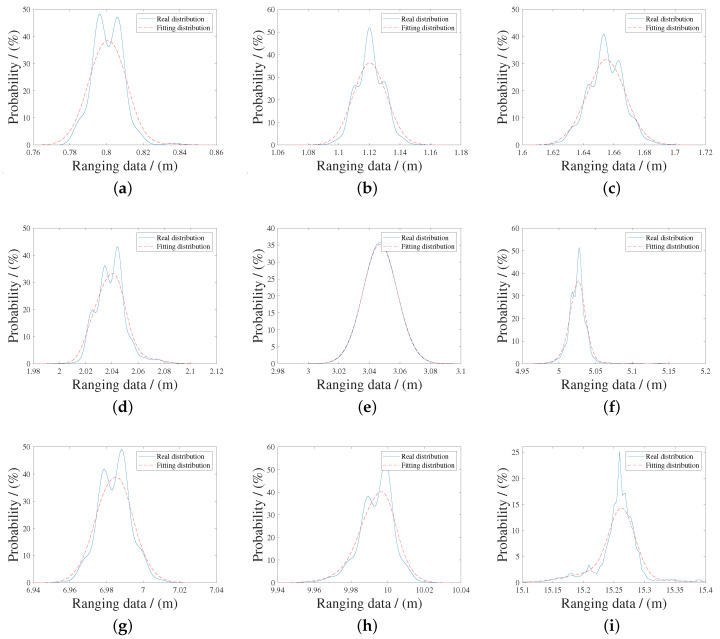

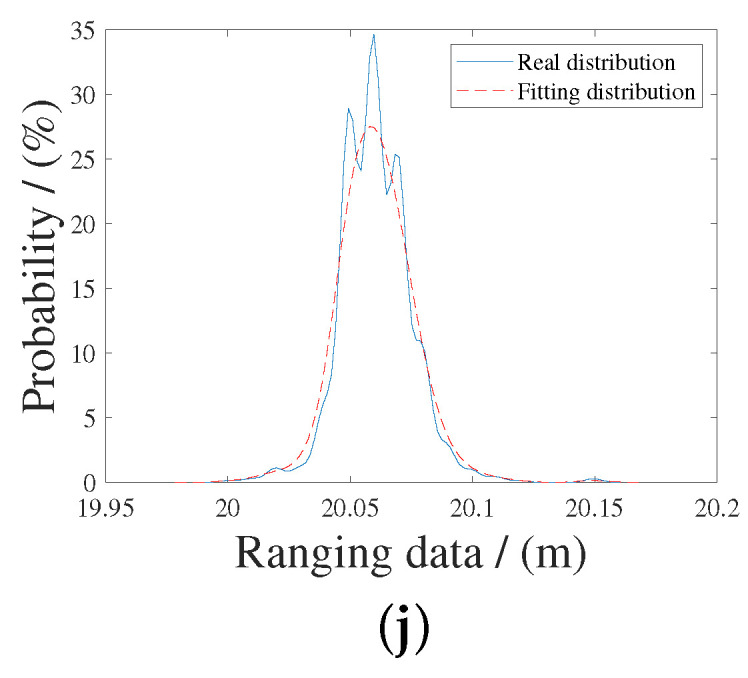
The probability of ranging measurements is shown, when distances are (**a**) 0.7 m, (**b**) 1 m, (**c**) 1.5 m, (**d**) 2 m, (**e**) 3 m, (**f**) 5 m, (**g**) 7 m, (**h**) 10 m, (**i**) 15 m, (**j**) 20 m. At every experiment, the probability distribution is approximately Gaussian distribution, but there is a deviation between the maximum probability value and the true value, shown in Table 1.

Let the true value of the distance between the tag and the anchor be $x_{true}$ and the measured value be $x_{m}$. 1500 ranging experiments were performed at 10 different $x_{true}$ in total. Table 1 shows the results of ranging experiments, got by (1), and Figure 5 shows the probability distribution of various distances, which are approximately Gaussian distributions.

**Figure 6:**
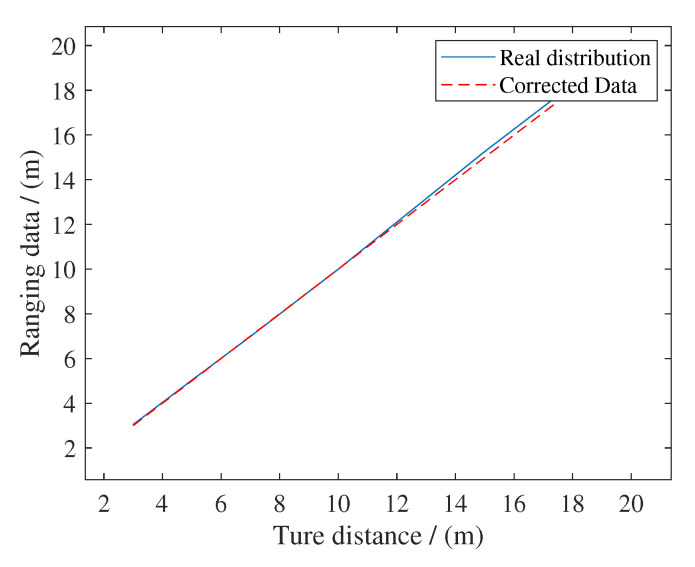
Fitting results from the distance between 3 m to 20 m. Corrected measurements show better linearity and are closer to the true value.

**Figure 7:**
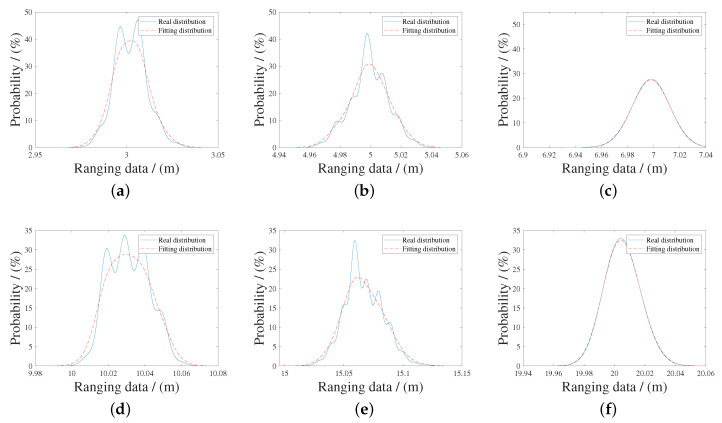
The probability of corrected ranging measurements is shown when distances are (**a**) 3 m, (**b**) 5 m, (**c**) 7 m, (**d**) 10 m, (**e**) 15 m, (**f**) 20 m. At every experiment, the probability distribution is approximately Gaussian distribution, and there is a smaller deviation between the maximum probability value and the true value, shown in Table 2.

Due to the geometric relationship and the influence of terrain, the distance between the robot and a certain anchor is mostly in the range of 3 m–20 m. Therefore, we can find the relationship between $x_{true}$ and $x_{m}$, shown in (22). Table 2 shows the results and Figure 6 shows fitting results from the distance between 3 m to 20 m. Figure 7 shows the probability distribution of this group of ranging values

(22) $$x_{true}=1.0172\;x_{m}-0.0745$$

where $x_{m}$ is the measured value between the tag and Anchor${}_{i}$ and $x_{true}$ is the corresponding true distances.

**Figure 8:**
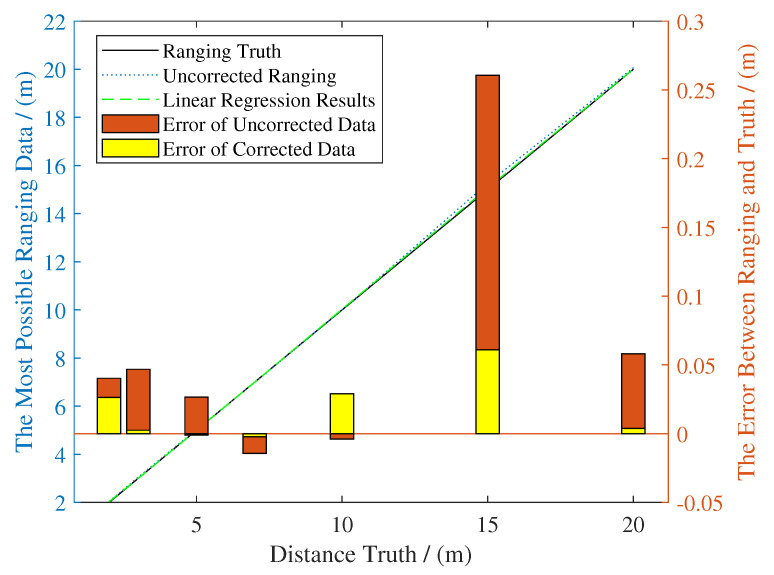
Comparison shows linear regression improves the accuracy of ranging, with error limited in 0.05 m.

Figure 8 shows the comparison results of two experiments. In the vicinity of general working range (3 m–20 m) of the robot, corrected ranging results is more accurate, especially when $x_{true}$ is 15 m (from 0.25 m to about 0.05 m).

### 3.3. Global Localization

**Figure 9:**
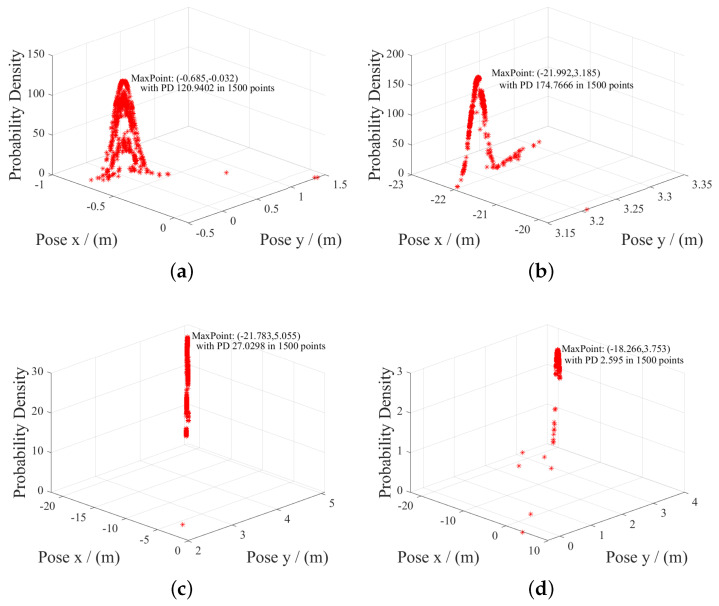
The probability distributions of poses is shown when UAPF conducts global localization in four positions, (**a**) ($x_{true},y_{true}$) = (−0.6,0), ($x_{true},y_{true}$) = (**b**) (−22,3), ($x_{true},y_{true}$) = (**c**) (−21.7,5), (**d**) ($x_{true},y_{true}$) = (−18,3.7), which obey Gaussian distributions and is consistent with some theoretical hypotheses.

**Figure 10:**
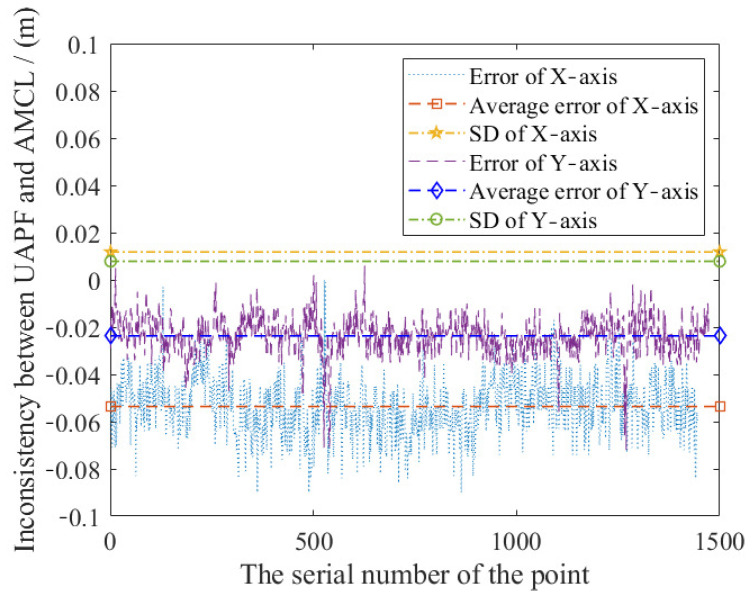
The stability in both X and Y directions. Average errors are both within 0.05 m and the standard deviation (SD) is less than 0.01 m.

Global positioning accuracy is measured to figure out whether the correction of ranging useful, in which Probability Density Estimation (PDE) is conducted. Figure 9 shows the probability distribution of ranging-based poses. Most of the measurement points are near the true coordinates. Figure 10 shows that the deviation of ranging-based localization is within 0.2 m in both X and Y directions.

**Figure 11:**
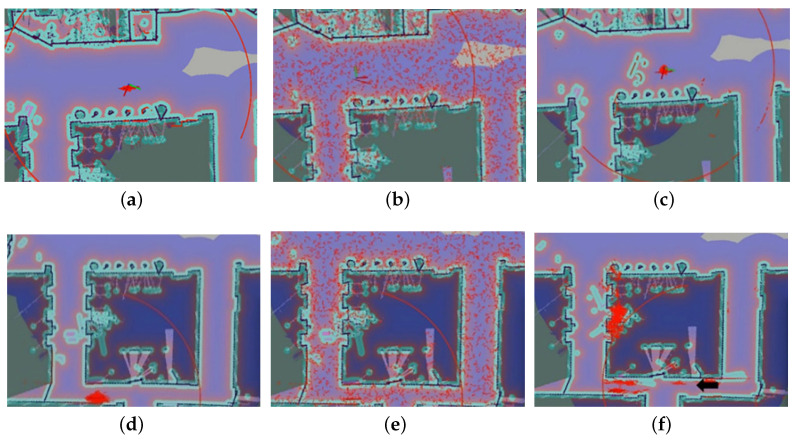
Comparison of global localization between AMCL and UAPF. (**a**) The initial pose of UAPF. (**b**) Global localization with UAPF. (**c**) Result of global localization using UAPF. (**d**) The initial pose of AMCL. (**e**) Global localization with AMCL. (**f**) Result of global localization using AMCL.

For PF-based global localization, better performance generally comes with more particles and bigger covariance. However, in large scenarios, this relation becomes more blurred because of few features for scan matching. Besides, more particles mean more consumption cost. Table 3 shows the time required for UAPF to achieve global localization when the number of particles is less than 1000. In the course of 10 experiments, the average time is 2.1 s, compared to more than 90 s with AMCL shown in Table 4, where false convergence occurs with the number of particles almost 10,000 (shown in Figure 11f).

### 3.4. Robot Kidnap Recovery

**Figure 12:**
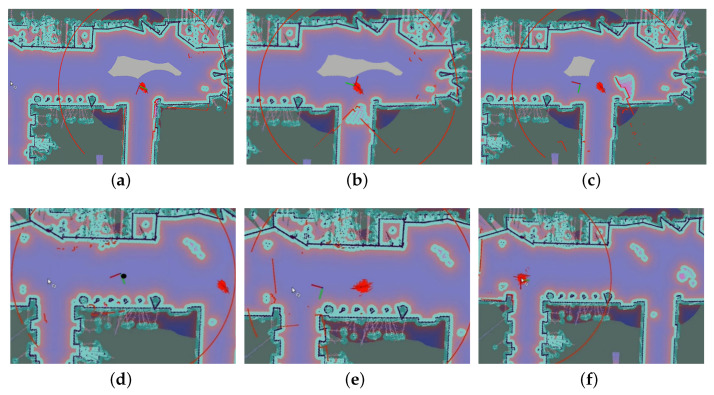
How UAPF achieves robot kidnap recovery. (**a**) Initial pose. (**b**) Rotating without odometry information. (**c**) Moving without odometry information. (**d**) First pose recovery. (**e**) The odometry is activated. (**f**) Second pose recovery is triggered, particles swarm is converged to true pose.

Intuitively, it’s easy to achieve recovery from robot kidnapping if the particles swarm has more particles and bigger covariance, which could cost more. Therefore, in this subsection, the number of particles is from 500 to 1000 for UAPF, and from 5000 to 10,000 for AMCL. To simulate a robot kidnap, we move the robot without data of odometry. Figure 12b,c express the situation where no kidnap recovery is performed because KNP is higher than the threshold. Figure 12c shows that when the robot moves, PF-based localization can’t achieve robot kidnap detection in real time, making KNP and the uncertainty of PF-based localization increase. Then, in Figure 12d, UAPF achieves a robot kidnap recovery. The odometry is enabled in Figure 12e, when the PF-based localization works normally but there is still some inconsistency between two pose distribution. Figure 12f shows the results of the kidnap recovery. When the odometry, lidar, and UWB work simultaneously, an accurate localization can be achieved.

### 3.5. Pose Tracking

**Figure 13:**
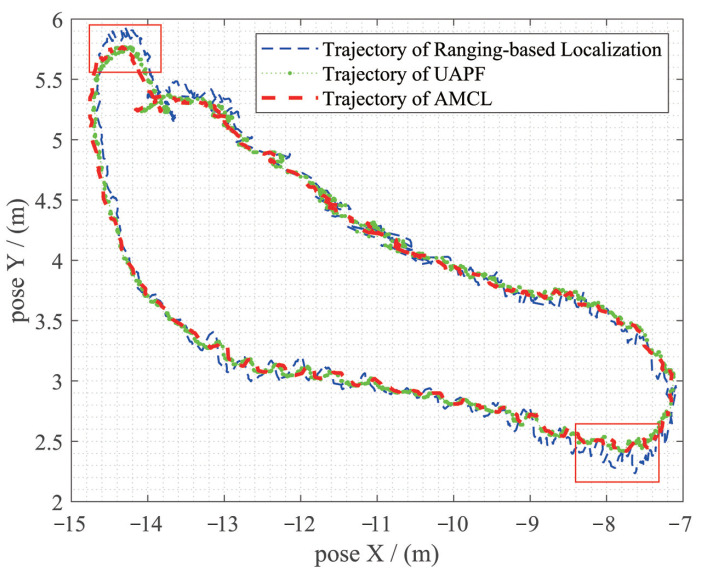
Trajectories of ranging-based localization, UAPF and AMCL. In general, the three have similar performance in accuracy. The two red rectangles show that UAPF could correct the instability of ranging-based localization.

Figure 13 shows the trajectories of single ranging-based localization, Adaptive Mento Carlo Localization and UAPF. The trajectory of individual ranging-based localization is more unstable while the trajectory of UAPF is closer to AMCL (as compared). The two red rectangles show that when a huge bias (about 0.2 m) of ranging-based localization exists, UAPF has the analogous performance to AMCL.

## 4. Discussion

In this paper, we presented an indoor localization method for open scenarios with few features. Ranging-based localization provided the initial pose for first global localization, and then pose fusion was conducted as the basis of normal pose tracking. Moreover, we used PF-based localization to overcome noise from sensors. A novel criterion called KNP was introduced into UAPF to evaluate the possibility of robot kidnapping and the stability of localization together with the covariance of particles swarm. Experiments in a real-world situation indicated UAPF could achieve robot kidnap recovery in less than 2 s and position error is less than 0.1 m in a hall of 600 m${}^{2}$.

In Section 3, we compared our method with AMCL, because it’s state of the art PF-based indoor localization method using lidar and odometry. Table 1 and Table 2 indicated that the regression function (22) was suitable for experimental scenario and Figure 8 showed intuitively how linear regression improves the accuracy of ranging.

Table 3 and Table 4 expressed time to achieve global localization with UAPF and AMCL separately. Table 5 compared the accuracy and time used to conduct the recovery from robot kidnapping. As mentioned above, the number of particles used in UAPF was from 500 to 1000 and in AMCL was from 5000 to 10,000. In this situation, UAPF could still conduct global localization in less than 3 s on average, much less than AMCL, illustrating the efficiency of UAPF. Figure 11c,f showed results of global localization. Figure 12a–f expressed the process of robot kidnap recovery. Trajectories of different localization methods were shown in Figure 13, illustrating UAPF could achieve analogous performance to AMCL, much stable than single ranging-based localization method, which restricted further improvement of accuracy.

In the future, the instability of ranging-based localization will be improved, and more sensors such as RGBD will be added to UAPF and make it a universal localization method. Vision-based localization will play an essential role when the robot is in an NLOS environment, lack of ranging information transferred by UWB.

## 5. Conclusions

In this paper, a UWB aided Particle Filter Localization method is designed to solve the problem of robot kidnap recovery and global localization in open scenarios with few features. Integrating odometry, lidar and UWB, UAPF achieves adaptive pose error compensation, as well as robust robot kidnap detection and recovery. Besides, for reliable pose tracking, pose fusion is utilized to combine PF-based localization and ranging-based localization, returning a relatively accurate pose. The probability of robot kidnap is estimated according to KNP and the uncertainty of particle swarms, and pose recovery is triggered based on the latest ranging-based pose, eliminating accumulated errors of UAPF. To improve localization accuracy, a revised ranging model based on statistical analysis is summarized from extensive experiments. The results show UAPF can achieve robot kidnap recovery in less than 2 s and position error is less than 0.1 m in a hall of 600 m${}^{2}$, much more efficient than the currently prevalent lidar-based localization.

## Figures and Tables

**Table 1:** Results of the ranging experiment.

$x_{\mathrm{true}}$ (m)	Average of $x_{m}$ (m)	Error (m)	Standard Deviation (m)	The Most Probable Value (m)
0.70	0.8007	0.1007	0.0078	0.80045
1.00	1.1204	0.1204	0.0085	1.1205
1.50	1.6548	0.1548	0.0112	1.6544
2.00	2.0394	0.0394	0.0109	2.0403
3.00	3.0471	0.0471	0.0055	3.0468
5.00	5.0254	0.0254	0.0117	5.0266
7.00	6.9843	0.0157	0.0082	6.9856
10.00	9.9938	0.0062	0.0087	9.9961
15.00	15.2645	0.2645	0.0562	15.2606
20.00	20.0607	0.0607	0.0147	20.0580

**Table 2:** Results of experiments after correction

$x_{\mathrm{true}}$ (m)	Average of $x_{m}$ (m)	Error (m)	Standard Deviation (m)	The Most Probable Value (m)
3.00	3.0023	0.0023	0.0082	3.0025
5.00	4.9993	0.0007	0.0121	4.9989
7.00	6.9979	0.0021	0.0074	6.9978
10.00	10.0313	0.0313	0.0108	10.0291
15.00	15.0668	0.0668	0.0158	15.061
20.00	20.0051	0.0051	0.0071	20.0039

**Table 3:** Time to achieve global localization of UAPF.

Experiment number	1	2	3	4	5	6	7	8	9	10
Convergence time (s)	2	2	1	2	1	6	2	2	2	1

**Table 4:** Time to achieve global localization of AMCL.

Experiment number	1	2	3	4	5	6	7	8	9	10
Convergence time (s)	117	83	74	72	83	66	102	97	132	85

**Table 5:** Performance of robot kidnap recovery with UAPF and AMCL

Localization Methods	Time (s)	Max Error (m)	Number of Particles	Processor
AMCL	90	2.00	5000–10,000	i3 CPU
UAPF	3	0.15	500–1000	i3 CPU
